# Secretory Peptides as Bullets: Effector Peptides from Pathogens against Antimicrobial Peptides from Soybean

**DOI:** 10.3390/ijms21239294

**Published:** 2020-12-05

**Authors:** Yee-Shan Ku, Sau-Shan Cheng, Aisha Gerhardt, Ming-Yan Cheung, Carolina A. Contador, Lok-Yiu Winnie Poon, Hon-Ming Lam

**Affiliations:** 1 Centre for Soybean Research of the State Key Laboratory of Agrobiotechnology and School of Life Sciences, The Chinese University of Hong Kong, Hong Kong; ysamyku@cuhk.edu.hk (Y.-S.K.); susansaushan@gmail.com (S.-S.C.); aisha.gerhardt@gmail.com (A.G.); cheungmy@cuhk.edu.hk (M.-Y.C.); ccontador@cuhk.edu.hk (C.A.C.); winniepoon1017@gmail.com (L.-Y.W.P.); 2 Centre for Organismal Studies (COS), Heidelberg University, Im Neuenheimer Feld 230, 69120 Heidelberg, Germany

**Keywords:** soybean, pathogen, signaling, effector, secretory peptide, antimicrobial, endophyte, rhizospheric microbe, biopesticide

## Abstract

Soybean is an important crop as both human food and animal feed. However, the yield of soybean is heavily impacted by biotic stresses including insect attack and pathogen infection. Insect bites usually make the plants vulnerable to pathogen infection, which causes diseases. Fungi, oomycetes, bacteria, viruses, and nematodes are major soybean pathogens. The infection by pathogens and the defenses mounted by soybean are an interactive and dynamic process. Using fungi, oomycetes, and bacteria as examples, we will discuss the recognition of pathogens by soybean at the molecular level. In this review, we will discuss both the secretory peptides for soybean plant infection and those for pathogen inhibition. Pathogenic secretory peptides and peptides secreted by soybean and its associated microbes will be included. We will also explore the possible use of externally applied antimicrobial peptides identical to those secreted by soybean and its associated microbes as biopesticides.

## 1. Introduction

Soybean (*Glycine max* L.) is a major legume crop produced and consumed worldwide due to its rich protein and oil contents [1,2]. Its uses range from nutritious human food and animal feed to industrial products [3]. The high demand for it has resulted in the expansion of arable land dedicated to soybean cultivation and the production of more than 340 million tons worldwide in 2019 [1,4]. As with other crops, soybean production is subject to environmental stresses which limit growth, development, and/or productivity [5]. These environmental stresses can be abiotic or biotic, or a combination of both. Abiotic stresses are related to the physical environment including weather, nutrients, and soil conditions [1,5]. On the other hand, biotic stresses could be due to plant diseases, insect pests, and competition from weeds [1,6]. Among these, plant diseases and pests have the most significant negative impacts on yield and quality of crop production [7,8]. These cause economic losses and reduce global food/feed security [7,8].

In the natural environment, plants are constantly challenged by biotic stresses, including insect and pathogen attacks. The physical damages caused by insect bites usually make the plants vulnerable to pathogen infection, which causes diseases. Plant diseases can be caused by different types of pathogens including fungi, oomycetes, bacteria, viruses, and nematodes [9]. These pathogens infect and attack all parts of the plants from roots, stems, and leaves to pods and seeds. The economic damages caused by plant diseases depend upon the type of pathogen, plant tissue affected, number of plants affected, attack severity, host plant resistance and susceptibility, plant stress level, and plant developmental stage [10]. In 2019, the yield losses of soybean due to pathogens and pests were estimated to be 21.4% globally [2]. Efforts have been made to improve to understand the underlying mechanisms of soybean—pathogen. Such efforts include the search of useful genetic materials from different soybean germplasms including wild soybean, which has been reported to be more disease-resistant compared to cultivated soybean [11,12]. A number of genes which are related to disease resistance and metabolism were found to be present in wild soybeans but not cultivated soybeans [13,14]. In this review, we summarize the currently available information, which is mostly from cultivated soybeans.

Soybean pathogens include fungi, oomycetes, bacteria, viruses, and nematodes [9]. Among these pathogens, the mechanisms of infection by fungi, oomycetes, and bacteria are similar. In contrast, viruses are intracellular pathogens and nematodes attack the plant by feeding on it [9]. In this review, we will focus mainly on the microbial pathogens. The attack by microbial pathogens and the defense by soybean plants usually involve the secretion of small molecules such as peptides. The effects of pathogenic peptides on soybean plants include the suppression of the transcription of defense-related genes [15] and the synthesis of defense-related hormones [16], as well as sudden death of the plant [17,18,19,20]. Soybean plants react by secreting antimicrobial peptides, which inhibit the growth of the pathogenic microbes. In addition, soybean endophytes and rhizospheric microbes are also able to secrete peptides to inhibit the growth of the microbial pathogens [21,22,23,24,25,26,27]. Many of these secreted peptides have been demonstrated to exhibit antimicrobial activities in vitro. Such properties hint at the possible application of soybean antimicrobial peptides for pathogen control.

## 2. Introduction to Soybean–Microbial Pathogen Interactions

### 2.1. Soybean–Fungus Interactions

Fungal plant diseases can infect seeds, foliage, stems, and roots, and can cause catastrophic epidemics on crop plants. However, infection and colonization patterns vary between fungal species. Those species that require a living host to survive are referred to as obligate biotrophs [28]. There are several soybean diseases caused by biotrophic pathogens. The most damaging among them is soybean rust (SBR) caused by *Phakopsora pachyrhizi* Syd. and P. Syd. [29]. *P. pachyrhizi* is a biotrophic fungus that infects leaf tissue in a wide range of host species in the family Fabaceae [30]. The fungus produces urediniospores, teliospores, and basidiospores, among which urediniospores have been associated with disease development [31]. *P. pachyrhizi* infects its host by direct penetration of the epidermal cell in the dikaryotic phase [32]. The infection of susceptible tissue in the host is initiated when urediniospore-derived germ tubes result in an appressorium [32]. During the infection process, the appressorium forms a funnel-shaped structure called an appressorial cone that is contiguous with the cell wall of the penetration hypha [30,31,32]. Hypha penetration causes the collapse of the host epidermal cell, signified by necrosis [32]. As the hypha grows through the epidermal cell, it reaches the intercellular space to form a septum and elongates to form the primary hypha and secondary hyphae. Finally, a first haustorium is formed in the host mesophyll cells to initiate the development of the rust colony. Dome-shaped eruptions are observed in the epidermis from which uredinia develop to produce more urediniospores [30,31,32]. The wind-blown spores can be rapidly spread in fields and cause early defoliation, leaf yellowing, and pod abscission [1,9]. Another fungal disease also caused by biotrophic pathogens is powdery mildew (*Erysiphe diffusa* (Cooke and Peck) U. Braun and S. Takam) [33].

Besides obligate biotrophs, fungal pathogens can also be classified into two other categories, based on their interactions with the host and their lifestyle, namely necrotrophs and hemibiotrophs [28]. Necrotrophic fungi actively kill host tissues before invading and colonizing host plants. Host cell death can result in serious damage to plant tissues, resulting in the death of whole plants or large portions of the plants. In general, the infection mechanism of necrotrophic fungal pathogens involves the attachment of conidia to the host plant [34]. After attachment, conidia germinate, and penetration may be achieved through a conidium-derived germ tube, followed by appressorium formation and enzymatic degradation of wounding sites [28,34,35]. Lytic enzymes involved in breaking down host tissues include oxidases, cutinases, and lipases for degrading the plant cuticle and waxy layers [28,36]. After gaining entry into the host plant, the fungal pathogen proceeds to degrade the underlying cells with lytic enzymes and cell wall-degrading enzymes, inducing necrotic lesions and accessing nutrients in the host cells [28]. Phytotoxic metabolites ranging from host-specific to broad-spectrum are commonly produced by necrotrophic pathogens to facilitate necrosis development in the host [35]. Fungal diseases caused by soil-borne necrotrophic fungi include sudden death syndrome (*Fusarium virguliforme*), foliar blight (*Rhizoctania solani*), charcoal rot (*Macrophomina phaseolina*), and brown stem rot (*Phialophora gregata* sp. *sojae*) [37,38,39,40]. Other diseases include cercospora leaf blight and purple seed (*Cercospora kikuchii*), brown spot (*Septoria glycines*), frogeye leaf spot (*Cercospora sojina*), and stem rot (*Sclerotinia sclerotiorum*) [41].

On the other hand, hemibiotrophic fungi represent a group of pathogens, such as those belonging to the genus *Colletotrichum*, that use sequential biotrophic and necrotrophic infection strategies to infect and colonize host plants. Anthracnose is a soybean disease that can infect soybean plants at any stage of development and can cause significant damage to seed quality and lower yield [42,43]. *Colletotrichum truncatum*, *C. coccodes*, *C. gleosporioides*, *C. graminicola,* and *Glomerella glycines* are some of the pathogen species associated with anthracnose [41]. *C. truncatum* is a seed-borne fungus that can survive in soybean debris [42]. The infection process begins with conidial germination on plant tissues. After the development of appressoria, hyphae penetrate to colonize the intracellular space. The necrotrophic phase of the infection initiates when the secondary hyphae emerge from the primary hyphae, causing the collapse of epidermal and mesophyll cells [17,28]. This causes extensive necrotic lesions where acervuli can be observed within two days after infection [42].

### 2.2. Soybean–Oomycete Interactions

Oomycetes are a group of eukaryotic pathogens that can cause serious damage to soybean production [18]. Oomycetes belong to the Kingdom Stramenopila, and despite not being classified as fungi, these organisms present several morphological and physiological traits in common with fungi [19]. Modes of infection by oomycetes can also be necrotrophic, biotrophic, or hemibiotrophic [20]. One of the most serious oomycete-related soybean diseases is caused by the soil-borne root rot pathogen *Phytophthora sojae* [44]. *P. sojae* is a hemibiotrophic pathogen that is responsible for damping-off of seedlings and root and stem rot in soybean [44,45]. The pathogen is dispersed mainly through soil-borne oospores and zoospores. Infection occurs when zoospores are released from sporangia and chemotactically attracted to nutrients released by host roots [46]. Zoospores attach to the plant surface and transform into adhesive cysts. Upon germination, a hypha is produced that penetrates the plant and a haustorium develops as the hypha branches to penetrate host cells [20,44]. This biotrophic interaction causes host cell necrosis [47]. Other oomycete pathogens affecting soybean include *Phytophthora* spp., *Pythium* spp., and *Phytopythium* spp. [45].

### 2.3. Soybean–Bacterium Interactions

As with fungi, bacteria also display different types of interactions with host plants. Bacterial pathogens are widespread and can reduce soybean productivity. Gram-negative bacteria associated with soybean diseases include *Pseudomonas syringae* pv. *glycinea* (bacterial blight), *Xanthomonas campestris* pv. *glycines* (bacterial pustule), *P. syringae* pv. *tabaci* (wildfire), and *Ralstonia solanacearum* (bacterial wilt) [48]. Gram-positive bacteria are responsible for diseases such as bacterial tan spot (*Curtobacterium flaccumfaciens* pv. *flaccumfaciens*) and fasciation (*Rhodococcus facians*) [48]. Bacterial blight and bacterial pustule are the most common bacterial diseases of soybean. Bacterial blight is most predominant in rainy seasons or high-humidity conditions [49]. The symptoms are easily observed on leaves of infected plants in the form of water-soaked and chlorotic lesions [49]. *P*. *syringae* pv. *glycinea* is a hemibiotrophic pathogen [50]. It infects aerial portions of plants (leaves and fruits) but rarely affects seeds [50]. The life cycle of this pathogen includes two phases: an initial epiphytic phase upon arrival on the surface of healthy plant tissue followed by an endophytic phase where bacteria colonize the apoplast by entering the plant through natural openings or wounds [49,51,52]. The invading bacteria cause host cell death, manifested as necrosis, in the infected tissues at the late stage of pathogenesis. Type III secretion systems are used to inject virulence factors into host cells to colonize the intercellular space [53].

Bacterial pustule causes premature defoliation and affects the size and seed quality [54]. *X. campestris* pv. *glycines* enters the plant through natural openings (stomata) or wounds and can infect soybean at any stage of plant development [48,54]. After entering the host plant through stomata, the bacteria invade and multiply within the substomatal chambers and intercellular space of the mesophyll [55].

### 2.4. Soybean–Virus Interactions

Soybean is susceptible to infection by numerous viruses either naturally or through artificial inoculation, but only a small number of viruses actually cause severe damage to soybean production. Soybean mosaic virus (SMV) is one of the most prevalent viral pathogens in soybean fields around the world. SMV belongs to the genus *Potyvirus* and its genome is a positive-sense single-stranded RNA of approximately 9.6 kb [56]. The genome encodes eleven proteins: P1 (potyvirus 1), HC-Pro (helper-component protease), P3 (potyvirus 3), PIPO (Pretty Interesting *Potyviridae* ORF), 6K1 (six kilo Dalton 1), 6K2 (six kilo Dalton 2), CI (cylindrical inclusion), NIa-VPg (nuclear inclusion a–viral protein genome-linked), NIa-Pro (nuclear inclusion a-protease), NIb (nuclear inclusion b), and CP (coat protein) [9,57]. Infected plants have reduced seed size and oil content as well as abnormal leaves (mosaic, mottle, and veinal chlorosis) [58]. The impact of the disease on crops depends on several factors including host genotype, virus strain, environmental conditions, and growth stage of the plant at infection [59,60]. The virus particle can be transmitted via infected seeds through vectors [61]. Over 30 aphid species transmit SMV in a nonpersistent manner including *Aphis glycines*, *A. fabae*, and *Acyrthosiphon pisum* [56]. HC-Pro and CP are associated with aphid transmission of virus particles, while seed transmission depends on the virus proteins P1, HC-Pro, and CP [62]. Other viruses affecting soybean include bean yellow mosaic potyvirus and soybean vein necrosis virus [56].

Common soybean diseases caused by fungi, oomycetes, bacteria, and viruses are summarized in Table 1.

## 3. Introduction to the Innate Immunity of Plants

When microbes invade plant cells, they could establish a symbiotic relationship with the plant or elicit the immune response of the plant [63]. Rhizobia and arbuscular mycorrhizae are common microbes that establish symbiotic relationships with soybean [64]. Soybean pathogens are summarized in Table 1. The balance between the promotion of beneficial symbiosis and the restriction of pathogenic attack is archived by signaling events between the microbe and the plant [63]. Receptors in the plant play important roles to mediate symbiotic responses or immune responses. Such balance between symbiosis and immunity has been summarized in a previous review [64]. In the following, signaling events that lead to immune responses in plants will be discussed.

The “gene-for-gene” model has been proposed to explain the resistance of plants to pathogens since the 1940s [65,66]. The model suggests that plants produce resistance (R) proteins to couple with avirulence (Avr) proteins from specific pathogen strains [65,66,67]. Such interactions result in gene-for-gene resistance [67]. *Avr* genes exist in microbes including fungi, oomycetes, bacteria, and viruses [68]. However, R protein and Avr protein compatibility is highly specific. Thus, such a mechanism for pathogen resistance is unable to confer a broad spectrum of resistance but can only be effective towards certain pathogens.

To explain the general defense response of plants against a broad spectrum of pathogens, a model originally used to explain how animals recognize pathogens was adopted to explain immunity in plants [69]. Elicitors that can trigger the immune response are termed pathogen-associated molecular patterns (PAMPs). Since PAMPs are conserved in both pathogenic and non-pathogenic microbes, PAMPs are also named as microbe-associated molecular patterns (MAMPs) [69]. The first identified PAMP is a conserved 22-amino acid epitope derived from bacterial flagellin (flg22) which is present across bacterial genera [70]. PAMPs can be recognized by a broad spectrum of plant species including tomato, tobacco, and rice [71]. Besides fragments of flagellin, EF-Tu (elongation factor thermal unstable), DNA, lipoproteins, lipopolysaccharides, and fungal chitin are also elicitors from microbes [72]. All these elicitors are recognized by pattern recognition receptors (PRRs) on the surfaces of plant cells. For instance, flg22 is recognized by a receptor kinase named FLS2 [73]. Upon recognition, a cascade of defense responses is elicited, including the generation of reactive oxygen species (ROS), calcium burst, activation of mitogen-activated protein kinases (MAPKs) and G-proteins, synthesis of salicylic acid (SA), jasmonic acid (JA), and ethylene (ET), followed by the resulting expressions of defense-related genes, such as *Pathogenesis-Related* (*PR*) genes. Such a response is known as the PAMP-triggered immunity system (PTI) [74,75].

**Figure 1:**
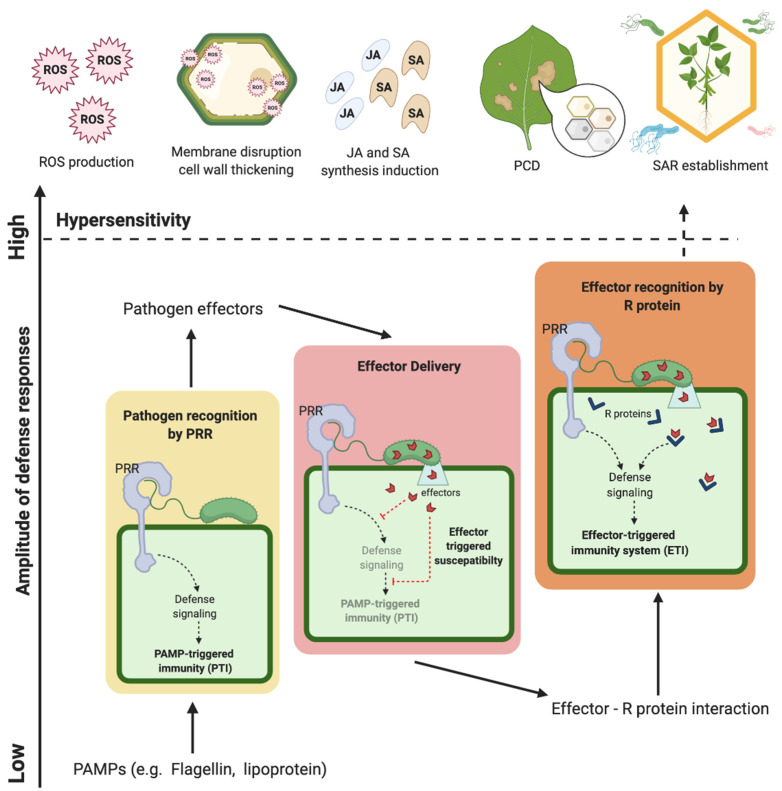
The overview of plant defense response. The interplay between pathogens, PTI (PAMP-triggered immunity), and ETI (effector-triggered immunity) is illustrated by the classic zig-zag model [79]. PAMPs (pathogen-associated molecular patterns) are recognized by PRRs (pathogen recognition receptors) and trigger the defense responses of plants. Some pathogens have developed effectors to neutralize PTI in plants. Consequently, plants develop ETI to counter the effects of pathogen effectors, and ETI is known as a boosted PTI response. ETI then triggers the onset of HR (hypersensitive response), in which ROS are produced to disrupt cell membrane, thicken the cell wall, induce JA (jasmonic acid) and SA (salicylic acid) production, and eventually, PCD (programmed cell death). The production of ROS also triggers the onset of SAR (systemic acquired resistance) to render the resistance to a broad spectrum of pathogens.

The PTI model suggests that plants generally exhibit resistance against a broad spectrum of microbes. However, some pathogens have developed effectors to interfere with the PTI in plants, which is known as effector-triggered susceptibility. From lower plants to higher, all plants face pathogen attacks. *R* genes are found from streptophyte algae to land plants [76]. However, it has been suggested that plants acquire diverse molecules such as PRRs to recognize various pathogens along evolution [77]. Some plants have evolved specific recognition mechanisms to detect and neutralize pathogenic effectors, hence achieving effector-triggered immunity (ETI) [78]. Such a recognition mechanism resembles the “gene-for-gene relationship” mentioned above [67]. PTI and ETI are integrated to explain the defense responses described as the “zigzag” model [79]. The responses of PTI and ETI eventually lead to systemic acquired resistance (SAR) [79]. Usually accompanied by programmed cell death (PCD) at the site of infection to restrict the spread of pathogens, ETI has been known as a boosted PTI response [79]. PCD is a part of hypersensitive response (HR), which occurs at the site of infection for restricting the spread of pathogens [79]. The production of ROS is a feature of HR. ROS cause the disruption of membrane, the thickening of the cell wall by crosslinking cell wall substances, the induction of jasmonic acid (JA) and salicylic acid (SA) syntheses, and eventually, PCD at the site of infection [80]. The production of ROS also triggers a signaling network to mediate the establishment of SAR [81]. SAR is not confined to the infected area but develops throughout the whole plant [82]. SAR enables the plant to be resistant to a broad spectrum of pathogens including pathogens which are originally infectious to the plant [82]. SAR is accomplished by the induction of pathogenesis-related (PR) proteins which have antimicrobial functions [83]. The overview of the defense response is illustrated in Figure 1.

## 4. Compatibility of Released Molecules from Plants and Pathogens Determine Disease Susceptibility

PTI is a non-host response of the plant and mainly relies on reinforcing physical barriers including the cuticle and the cell wall [83]. Physical damages of these barriers by various factors such as pests, herbivores, wind, or rainstorms create openings for microbial infection. It was found that not only elicitors derived from pathogens could trigger PTI—degraded molecules or endogenous peptides released by plant cells due to pathogen attacks could also trigger PTI. Such molecules originating from plants are called damage-associated molecular patterns (DAMPs) [83]. The first identified DAMP is systemin found in solanaceous plants [84]. Wounding due to insect bites leads to the production of a 200-amino acid prohormone, namely prosystemin, in the phloem parenchymal cells. Upon its synthesis, prosystemin is quickly cleaved into an 18-amino acid peptide, systemin, which is then transmitted through the vascular tissue to distal areas where it is perceived by the receptor SR160 in neighboring companion cells. The activation of SR160 results in the synthesis of jasmonic acid (JA), which induces the expression of defense-related genes such as those encoding proteinase inhibitors [84,85]. Besides systemin, examples of DAMPs include 18-amino acid hydroxyproline-rich glycopeptides (HRGPs) derived from a 165-amino acid precursor, 23-amino acid plant elicitor peptides (Peps) derived from a 92-amino acid precursor, oligogalacturonides (OGs) derived from a linear polymer of 1, 4-linked α-D galacturonic acid in the cell wall, and extracellular ATP (eATP), which are perceived by the receptors PEPR1 (in plant meristematic and differentiating zones), PEPR2 (in plant meristematic and differentiating zones), WAK1 (profoundly associated with plant cell wall), and DORN1 (in root apical meristem), respectively [86].

Whether a particular plant is susceptible to the attack by a particular microbe depends on the everlasting battle between the plant and the microbe in recognizing the pathogen or evading detection by the plant. For example, the soybean phytoalexin, glyceollin, which is synthesized in the isoflavonoid branch of the phenylpropanoid pathway in response to various pathogen attacks such as those by the fungal pathogen *Fusarium virguliforme*, can restrict colonization by avirulent pathogens. However, virulent pathogens, such as *Colletotrichum truncatum*, *Rhizoctonia solani*, *Cercospora sojina*, *Macrophomina phaseolina*, *Sclerotinia sclerotiorum*, and *Diaporthe phaseolorum* var. *meridionales* can secrete enzymes to degrade glyceollin and thus, overcome the basal defense response of soybean [87].

In Section 4.1 and Section 4.2, the battle of recognition and evasion between soybean and its pathogens will be discussed.

### 4.1. PAMP Sequence Polymorphism Influences Plant Susceptibility

Detection of conserved PAMP elicitor domains from different pathogens often involves the same receptor in the host. For instance, flg22, the conserved 22-amino acid peptide derived from the N-terminus of bacterial flagellin, is recognized by its cognate flagellin sensing 2 (FLS2) protein. The binding of flg22 to FLS2 promotes the hetero-dimerization with BRASSINOSTEROID INSENSITIVE 1-associated receptor kinase 1 (BAK1), which acts as the co-receptor of the C-terminus of FLS-bound flg22, leading to the activation of innate plant immunity against pathogens [73]. The recognition of flg22 by the soybean GmFLS2 (*Glycine max* FLAGELLIN SENSING 2) receptor enhances the phosphorylation of GmMPK3 and GmMPK6, and eventually, activates the downstream GmMAPK signaling pathway [88].

The amino acid sequence polymorphisms of effector proteins play an important role in the pathogenicity. For example, *Ralstonia solanacearum*, which causes bacterial wilt disease, is infectious towards more than 250 plant species including many legume species except soybean [89,90]. The broad host range of *R. solanacearum* results from the evolution of polymorphisms in the flg22 sequence compared to the flg22 in other pathogens such as *Pseudomonas syringae* and *P. aeruginosa*. The polymorphism in the flg22 of *R. solanacearum* strains hinders the recognition of flg22 by the FLS2–BAK1 receptor complex in susceptible hosts [90]. The common I21A mutation in flg22 disrupts the binding free energy of the FLS2/BAK1/flg22 complex and leads to the failure of the hosts to perceive flg22 [90]. However, soybean has also evolved polymorphic versions of the flg22 receptor to perceive flg22^Rso^ and shows increased defense response against *R. solanacearum* [90]. Examples of the polymorphisms include the amino acid substitutions at Q368 and R483 of GmFLS2b [90]. This suggests the importance of particular residues for facilitating flg22^Rso^ perception [90].

### 4.2. Post Translational Modifications of PAMPs Could Influence the Virulence

Other than the amino acid sequences, the post-translational modification patterns of PAMPs might also affect their perception by plants [91]. For example, the amino acid sequences of flagellin from *Pseudomonas syringae* pv. *tabaci* and *P. syringae* pv. *glycinea* are identical, but their abilities to induce a hypersensitive response in tobacco and soybean are different [91]. *Pseudomonas syringae* pv. *tabaci* and *P. syringae* pv. *glycinea* are virulent to tobacco and soybean, respectively. This different virulence is due to the glycosylation of the *P. syringae* pv. *glycinea* flagellin. *P. syringae* pv. *glycinea* flagellin became avirulent to soybean but was instead virulent to tobacco when it failed to be glycosylated [91]. Tobacco was originally a non-host to *P. syringae* pv. *glycinea* [91]. The change in virulence could be the result of the change in the biochemical properties of the flagellin molecule, such as hydrophilicity, as a result of the presence/absence of glycosylation [91,92].

### 4.3. Peptides Play Important Roles in the Defense Responses of Soybean

In response to the attack from pathogens and herbivores, other small signaling peptides similar to systemin have also been discovered in soybean [93,94,95,96]. A 12-amino acid peptide isolated from soybean leaf was found to be able to alkalinize the medium of a soybean cell suspension culture within 10 min [93]. It was suggested that the activity of this peptide resembles that of other peptidic defense signaling peptides in plants, but it is derived from an extracellular protease. Extracellular alkalinization has been known to contribute to fungal virulence [94]. Based on the amino acid sequence, it was proposed that the peptide is derived from a legume-specific subtilisin-like protease (subtilase) and was named GmSubPep. The subtilase was predicted to be secreted into the apoplast and then, cleaved to produce GmSubPep when it was in contact with fungal or bacterial components. It was proposed that GmSubPep is recognized by an unknown receptor and triggers defense responses including the induction of defense-related genes *achs* (*Chalcone synthase*), *PDR12* (a gene encoding pleiotropic drug resistance-type transporter), *Chib-1b* (a gene encoding PR-8 chitinase), and *CYP93A1* (encoding cytochrome P450) [93].

Following the identification of GmSubPep, other small peptides that are capable of triggering defense responses were also identified in soybean. Examples are GmPep914 (DHPRGGNY) and GmPep890 (DLPRGGNY). *GmPROPEP914* and *GmPROPEP89* are the genes that encode the precursors of GmPep914 and GmPep89, respectively [95]. *GmPROPEP914* and *GmPROPEP89* are highly expressed in roots [95]. As the addition of inactive GmSubPep analogs did not affect the abilities of GmPep914 or GmPep890 to alkalinize the media of cell suspension cultures, GmPep914 and GmPep890 were suspected to be recognized by receptors different from that of GmSubPep [95]. In addition, GmPep1, GmPep2, and GmPep3 were identified in soybean through a homology search against a family of eight Peps first reported in Arabidopsis. [96] GmPep1, GmPep2, and GmPep3 defend the soybean plant against bacterial and fungal pathogens. Additionally, these peptides reduce the reproduction of root-knot nematode (*Meloidogyne incognita*) and the soybean cyst nematode (*Heterodera glycines*) [96]. These form a family of peptides 23–36 amino acids long, harboring a glycine-rich motif: (S/G)(S)Gxx(G/P)xx(N). They are derived from the carboxyl termini of propeptide precursors (PROPEPs). It is found that GmPeps activate resistance against a broad spectrum of nematodes through the generation and accumulation of reactive oxygen species (ROS) at the apoplast, which then induces systemic resistance throughout the whole plant [96].

Evidence suggests the importance of peptides in these defense responses of soybean. Moreover, more and more reports suggest that the peptides involved in defense responses are secretory and exhibit antimicrobial activities. Antimicrobial peptides produced by plants, especially those by soybean, will be discussed in greater detail in Section 6 and Section 7.

## 5. Peptides Secreted by Soybean Pathogenic Microbes during the Attack

As discussed in Section 4, plants and their pathogens have co-evolved to form a dynamic relationship. Plant pathogens have evolved a myriad of ways to modulate their hosts’ immune systems to cause diseases. Small effector peptides secreted by pathogens have gained prominence in their roles in the pathosystems of important crop plants and their unwanted guests. Using a dual transcriptomics approach in *Nicotiana benthamiana* roots, a temporal gene expression profile during infection by *Phytophthora palmivora*, a tropical oomycete, was established [97]. Great variations in the gene expression patterns between the “early infection” and “late infection” time points were shown in the pathogen, whereas in the plant host, there was a more uniform response to the pathogen stress between these time points. In the report, four new putative RXLR (Arg-Xaa-Leu-Arg) effector-expressed (REX) proteins, namely REX1, REX2, REX3, and REX4, were identified from *P. palmivora*. The REX proteins shared a conserved RxLR sequence that facilitated entry into the host cell. Among the REX proteins, REX3 was shown to be able to impair host secretion processes [97]. Therefore, it was suggested that REX3 may promote infection by impairing host secretion processes [97]. This comprehensive study on a non-model representative of these oomycete pathogens laid the groundwork for similar research studies on related *Phytophthora* pathovars. In addition, this study provided insights into the infection mechanisms that have evolved in this species. For example, *P. sojae* causes root and stem rot in soybean and has evolved various mechanisms to subvert plant innate immunity by targeting the host’s defense reactions at different metabolic levels to ultimate suppression. Upon infection, *P. sojae* adheres to the host’s tissues, encysts, and forms germ tubes at the infection site. After entry into the plant, haustoria grow into the plant cells to extract useful compounds, while introducing effectors to cause virulence [44].

### 5.1. Pathogenic Effector Peptides Repress the Immune Responses of Soybean

Pathogens have often evolved a sophisticated desensitization mechanism to repress the immune responses by their plant host. For instance, *P. syringae* secretes AvrB through the type III secretion system (T3SS) to suppress flg22-induced callose deposition (a form of plant defense through thickening of the cell wall), suppress basal defense responses, and foster the progression of *Pseudomonas* sp. infection [98]. AvrB confers virulence on soybean plants which lack the cognate resistance gene *Rpg1-b*. Recognition of AvrB may activate the downstream immune response by interacting with the RPG1-b and GmRIN4 (RPM1-interacting 4) proteins in soybean [99,100,101]. In Arabidopsis, the phosphorylation of the RIN4 protein upon flg22 perception mediates Rpg1b-mediated resistance [102]. Although the aforementioned effects by *P. syringae* have not been reported in soybean, other effects on GmRIN4 by *P. syringae* have been shown. A cysteine protease AvrRpt2 acts as a T3SS effector protein from *P. syringae*, inactivates the GmRIN4 homolog, and blocks the recognition of AvrB by soybean [103]. Another T3SS-secreted cysteine protease, AvrPphB, suppresses immune system activation by blocking the phosphorylation of GmRIN4 at T198, the normal response to AvrB recognition, by the proteolytic cleavage of kinases that target GmRIN4 [104]. Thus, AvrPphB inhibits Rpg1-b activation and Rpg1b-mediated resistance by circumventing the host’s defense responses and aids in the multiplication of the bacterium in the host plant [104,105,106]. Another T3SS cysteine protease effector from *P. syringae*, HopZ1, promotes the degradation of an isoflavone biosynthesis enzyme, 2-hydroxyisoflavanone dehydratase (GmHID1) [107]. Upon bacterial infection, *GmHID1* expression is induced [107]. To counter-react for its survival, *P. syringae* adopts the proteolytic cleavage of GmHID1 by HopZ1 [107]. Daidzein production was reduced dramatically with the introduction of HopZ1 in soybean plant [108], thus affecting the phytoalexin production in soybean and resulting in the increased susceptibility to *P. syringae* infection.

### 5.2. Peptides Secreted by Soybean Pathogens Cause Sudden Death of the Host

Pathogens might secrete multiple effectors to achieve pathogenesis. Sudden death syndrome (SDS) of soybean is caused by *Fusarium virguliforme*, the root-infecting fungal pathogen that elicits distinct foliar symptoms after the onset of flowering [109]. Phytotoxic effectors from *F. virguliforme* have been suggested to be the inducers of the disease, symptoms of which include necrosis of leaves. Possible candidates of the effectors include ethylene-inducing-like proteins (NLP), necrosis-inducing secreted protein 1 (NIS1), and *F. virguliforme* toxin (FvTox1) [110,111,112]. FvTox1 has been considered a major virulence factor of *F. virguliforme* in soybean. Although the actual pathogenesis of *F. virguliforme* is unknown, it is proposed that multiple phytotoxins from *F. virguliforme* coordinate their actions to result in SDS. Inactivation of FvTox1 using FvTox1-specific antibodies enhanced foliar resistance to SDS [110,111]. It is speculated that FvTox1 might initiate the degradation of Rubisco and lead to the accumulation of ROS [110]. The accumulation of ROS then initiates foliar SDS-like symptoms [110]. FvNIS1 is another effector candidate involved in producing SDS foliar symptoms. Overexpression of the FvNIS1 using the SMV-mediated transient expression system mimics the SDS foliar symptoms observed in field grown soybean [112]. The magnesium dechelatase encoded by the soybean *STAY-GREEN* gene (*GmSGR1*) was hypothesized to be the target of phytotoxins to develop SDS foliar symptoms [113]. It was proposed that GmSGR1 initiates the SDS foliar symptoms by removing the magnesium ion from chlorophyll a in the first step of chlorophyll degradation and leaf senescence [114].

### 5.3. Effector Peptides Secreted by Soybean Pathogens Influence the Epigenetics of the Host

#### 5.3.1. Effects on Histone Modification

Pathogen effectors act at the epigenetic level in the soybean host to modulate or suppress the widespread transcriptomic changes occurring during plant defense responses in their favor. One example of this is the *P. sojae* effector, PsAvh23, which inhibits the acetylation of lysine 9 of histone H3 (H3K9) in soybean and thereby suppresses the expression of defense genes. Spt-ADA-Gcn5-Acetyltransferase (SAGA) is a prototypical nucleosome-acetylating modification complex in soybean, composed of General Control Non-depressive 5 (GCN5), which is the catalytic subunit, and Alteration/Deficiency in Activation 2 (ADA2), which is the regulatory subunit. SAGA mediates H3K9 acetylation, which results in transcriptional activation. PsAvh23 acts by competing with GmGCN5 for the binding to ADA2 and thereby, decreasing the level of H3K9 acetylation, resulting in the repression of defense-related genes and thus, increasing the susceptibility of the plant to the infection [15].

Another *P. sojae* effector, PsAvh52, that acts on the epigenetics of soybean, has also been identified [115]. It was shown that this effector interacts with a putative soybean acetyltransferase, GmTAP1 (*Glycine max* acetyltransferase), which is normally localized in the cytoplasm. However, transient co-expression experiments in *Nicotiana benthamiana* showed that upon the introduction of PsAvh52, GmTAP1 was translocated to the nucleus, where it leads to the acetylation of histones, and therefore, increased the transcription of key susceptibility genes in *G. max.* In this manner, the *P. sojae* effector PsAvh52 makes use of the plant’s own enzyme, and by translocating it into the nucleus, modulates transcription levels, and increases the susceptibility of the plant to infection [115].

#### 5.3.2. Effects on mRNA Regulation

Targeting the regulation of mRNAs from the plant’s immune responses is another strategy *Phytophthora* has evolved. As part of its virulence repertoire, *P. sojae* secretes the effector *Phytophthora* Suppressor of RNA Silencing 1 (PSR1) that targets the plant PSR-1 Interacting Protein 1 (PINP1). PINP1 contains an evolutionarily conserved RNA helicase domain. It is suggested to be involved in RNA silencing, since the silencing of *PINP1* using artificial miRNAs caused a widespread change in the small RNA landscape (including the numbers of both miRNAs and siRNAs), similar to the landscape observed when PSR1 is present in the wild type plant. Both interactors are nucleus-localized, and PINP1 was shown to positively regulate plant defense by possibly promoting miRNA processing, which is interrupted by PSR1 to enhance disease susceptibility. It was suggested that PINP1 is involved in miRNA and siRNA biogenesis by possibly promoting the accumulation or function of Dicer-like 1 (DCL1), an RNase III-like enzyme that processes pri-miRNAs into pre-miRNAs to produce miRNAs. Hence, it was suggested that PSR1 directly targets PINP1 to obstruct small RNA accumulation pathways and promote disease [116].

*P. sojae* was also found to express the avirulence effector PsAvr3c, capable of modulating host pre-mRNA splicing to subvert plant immunity. PsAvr3c binds to the newly identified serine/lysine/arginine-rich proteins of *G. max* (GmSKRPs) and stabilizes them by a yet-unknown mechanism, thus preventing their degradation. GmSKRP1/2 function as regulators of the spliceosome complex and their stabilization by PsAvr3c leads to splicing variations in the host, ultimately leading to increased host susceptibility to *P. sojae* infection. Therefore, GmSKRP1/2 are considered negative regulators of soybean immunity by interfering with the process of mRNA splicing in the plant [117].

#### 5.3.3. The Possible Roles of lncRNAs in Transcription Regulation

The regulatory actions of long non-coding RNAs (lncRNAs) during pathogenesis or symbiotic events have been receiving more attention since it has been observed that some lncRNA transcripts are, in contrast to their names, translated into active small peptides with putative functions. A comprehensive study on lncRNAs in *P. sojae* was conducted and it showed a transcriptional correlation between lncRNA loci and their neighboring effector genes [118]. The involvement of lncRNAs such as those in *P. sojae* seems to be crucial during stage-specific biotrophy [118]. The regulatory roles of lncRNAs as drivers of effector secretion during pathogenesis should not be underestimated, and further research will certainly give fascinating results on this additional regulatory layer of disease promotion.

### 5.4. Effector Peptides Secreted by Soybean Pathogens Affect Phytohormone Biosynthesis in the Host

Phytohormone signaling is a key player in plant defense against pathogens, which was extensively reviewed [119]. A newly identified *P. sojae* effector, PsAvh238, directly targets the Type-2 1-aminocyclopropane-1-carboxylate synthase of *G. max* (Type 2 GmACS), an enzyme involved in ethylene biosynthesis. By inhibiting ethylene production, this *P. sojae* effector directly tackles the plant’s hormone signaling crosstalks aimed at defense against pathogens, hence disrupting plant immunity and causing disease, adding yet another layer to the strategy for pathogenesis by this oomycete [120].

**Figure 2:**
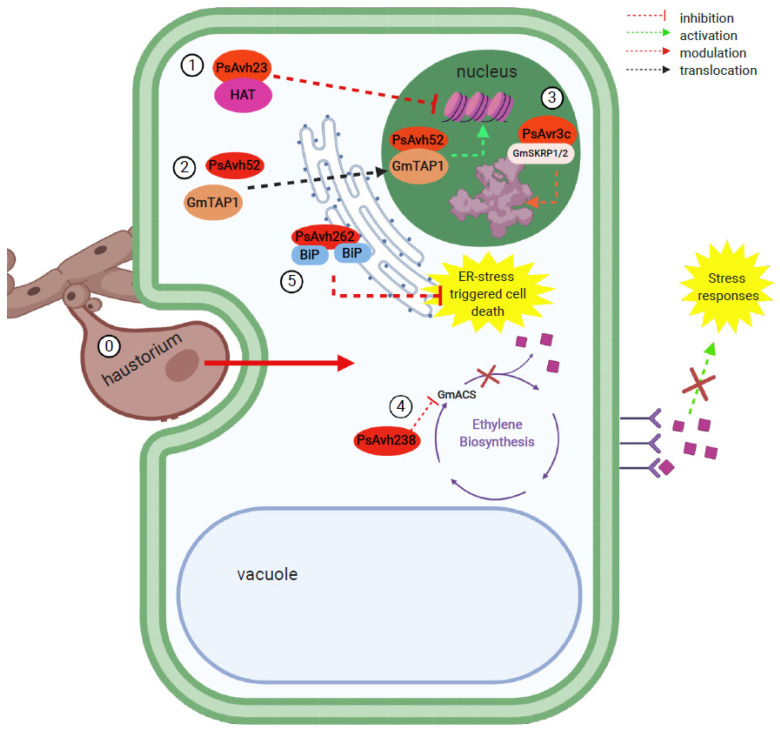
Representation of a selection of recently reported virulence peptides involved in the infection of plant cells by *P. sojae*. This figure shows a snapshot of the widespread metabolic changes induced by this oomycete as a result of the infection. (0) The haustorium penetrates soybean tissues and secretes effector peptides close to and into the plant cells [44]. (1) PsAvh23 binds to the regulatory subunit of the H3K9 histone acetyltransferase (HAT), preventing it from associating with the catalytic subunit and therefore, suppressing defense gene activation [15]. (2) PsAvh52 translocates the putative transacetylase protein GmTAP1 to the nucleus, leading to the acetylation of core histones and the upregulation of plant susceptibility genes [115]. (3) PsAvr3c stabilizes the spliceosome-associated protein GmSKRP1/2, causing changes in host pre-mRNA splicing, thus impairing plant immunity [117]. (4) PsAvh238 interacts with a type-2 aminocyclopropane-1-carboxylate synthase (GmACS), a key enzyme in ethylene biosynthesis, which disrupts ethylene signaling and therefore, impairs pathogen-induced stress responses in the plant host [120]. (5) PsAvh262 suppresses ER stress-triggered cell death by stabilizing luminal binding immunoglobulin proteins (BiPs), and therefore, promoting infection [121].

Examples of recently identified *Phytophthora* spp. effector peptides and their infection mechanisms are summarized in Table 2, and some of the infection mechanisms are illustrated in Figure 2.

## 6. Plant Antimicrobial Peptides

### 6.1. Introduction to Plant Antimicrobial Peptides

The presence of small peptides in plant cells has been supported by increasing evidence from genomic, transcriptomic, and proteomic research studies [16,127,128,129,130]. The small peptides could be intragenic [127], encoded by genes consisting of one or two exons [16,128] or encoded by lncRNAs [130]. These small peptides play important roles in signaling and defense [131]. The small peptides with antimicrobial functions are termed antimicrobial peptides (AMPs) [132,133,134]. The structural features and functions of plant AMPs have been summarized in previous reviews [132,133,134]. Briefly, plant AMPs are usually positively charged at physiological pH due to the high percentage of positively charged amino acid residues. The overall positive charge of AMPs facilitates the penetration of AMPs into the microbial cells through the cell wall, which is negatively charged. Plant AMPs may be expressed constitutively or induced upon pathogen attack [133]. The functions of plant AMPs include the disruption of the membranes of microbial cells and the induction of aggregation of bacterial cells [133]. AMPs are derived from precursor proteins which are cleaved [134]. Plant AMPs have been found in various plant tissues including seeds, pods, fruits, leaves, floral tissues, tubers, and roots [132]. Small cysteine-rich AMPs are produced by multicellular organisms for defense purposes [135]. Based on the tertiary structure of the peptides, AMPs are categorized into several groups, including defensins, thionins, lipid transfer proteins, cyclotides, and snaking [132]. Defensins are common among eukaryotes [135]. However, some AMPs are unique to the plant kingdom, such as thionins, lipid transfer proteins, and snaking [135]. By homology motif search of small putative secretory cysteine-rich peptides (CRPs), genes encoding defensins, thionins, lipid transfer proteins, snaking, and other antimicrobial peptides, including protease inhibitors and pollen allergens, were identified in the model plants *Arabidopsis thaliana* and *Oryza sativa* [135]. It was estimated that genes encoding CRP-like proteins account for 2–3% of the gene repertoires of both *A. thaliana* and *O. sativa* [135].

### 6.2. Nodule-Specific Cysteine-Rich (NCR) Peptides Are AMPs Unique to Certain Legumes

Legumes interact with rhizobia to form nitrogen-fixing nodules. Inside the nodules, rhizobia exist in the form of bacteroids, which are elongated, polyploid, and unable to carry out cell division [136]. It has been proposed that the occurrence of bacteroids in legumes is mediated by nodule-specific cysteine-rich (NCR) peptides [137,138]. NCR peptides could induce symptoms of terminal differentiation in rhizobial cultures [137]. It was also suggested that the root cells in the legume host are able to recognize yet-unidentified signals from the rhizobia and secrete NCR peptides to selectively inhibit incompatible rhizobia [139].

NCR peptides were first reported as a novel protein family in the transcriptomic study of *Medicago truncatula* nodules [140]. In *M. truncatula*, a group of nodule-specific, secretory peptides consisting of 60–90 amino acids were identified as NCR peptides [140]. By amino acid sequence alignment, it was found that NCR peptides from *M. truncatula*, pea, broad bean, *Garra orientalis*, and white clover share conserved domains [140]. Later, by position-specific iterated BLAST searches, transcripts predicted to be encoding NCR peptides were also identified in other legumes within the inverted repeat-lacking clade (IRLC), including *Glycyrrhiza uralensis*, *Onobrychis viciifolia*, *Oxytropis lamberti*, *Astragalus canadensis*, *Cicer arietinum*, *Ononis spinosa*, *Pisum sativum*, and *Medicago sativa*. *NFS1* (*Nitrogen fixation specificity 1*) from *M. truncatula* encodes an NCR peptide that promotes the cell death of *Sinorhizobium meliloti* Rm41, which is the rhizobium strain that forms non-functional nodules with *M. truncatula* [139,141]. Chemically synthesized NCR335 and NCR247, which are encoded in the *M. truncatula* genome, inhibited the growth of *S*. *meliloti* [142]. NCR335 and NCR247 were also found to be able to kill pathogenic bacteria, including Gram-negative and Gram-positive ones which infect animals and plants [142]. NCR335 induced complete cell disruption in *Salmonella enterica* (Gram-negative) while NCR247 led to extensive budding on the surfaces of *S. enterica* cells [143]. Both NCR335 and NCR247 led to morphological changes in *L. monocytogenes* (Gram-positive) cells while NCR335 caused cell leakage in *L. monocytogenes* [143]. Besides NCR335 and NCR247, 17 more NCR peptides encoded in the *M. truncatula* genome were found to be able to kill *Candida albicans*, which is a human fungal pathogen [144].

**Figure 3:**
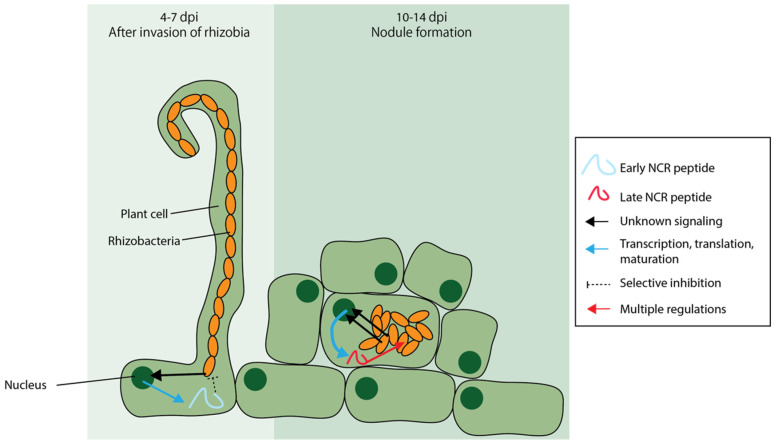
The secretion mechanism and modes of action of nodule-specific cysteine-rich (NCR) peptides. NCR peptides secreted at 4–7 days post inoculation (dpi) with rhizobia and at 10–14 dpi are classified as early and late NCR peptides, respectively [145]. The host root cells recognize unknown signals from the rhizobia and secrete NCR peptides, which may selectively inhibit incompatible rhizobia [139] and mediate bacteroid formation [137,138].

NCR peptides were previously only found in inverted repeat-lacking clade (IRLC) legumes [137]. However, later on, it was also reported that the Dalbergioid-clade legumes maintain bacteroids by a mechanism similar to that in IRLC legumes, owing to convergent evolution [138]. NCR peptide-like proteins have been found in the nodules of *Aeschynomene* spp., which belong to the Dalbergioid clade [138]. The mechanisms of secretion and functions of NCR peptides are illustrated in Figure 3. Recently, transcripts encoding NCR peptide-like proteins were also found in soybean, which possesses the inverted repeats [129]. These NCR peptide-like proteins in soybean will be discussed in Section 7.1.

## 7. AMPs Employed by Soybeans to Defend against Microbial Pathogens

The knowledge on AMPs employed by soybeans to defend against microbial pathogens has been limited. Nevertheless, currently available findings suggest that soybean plants as well as their associated microbes can secrete AMPs. The currently available information of characterized soybean AMPs is summarized in Table 3. The currently available information of soybean-associated microbes that exhibit antimicrobial activities is summarized in Table 5.

### 7.1. Soybean AMPs

By comparing the AMP sequences from the Antimicrobial Peptide Database, a collection of antimicrobial peptides from bacteria, archaea, protists, fungi, plants, and animals [146], with the genome sequence of *G. max*, putative intragenic antimicrobial peptides (IAPs) were identified [147]. Two putative IAPs—Gm0025x00667(75–100), which is a fragment of flavonoid 3-hydroxylase, and Gm0026x00785(77–103), which is derived from lipoate-protein ligase B—were selected for functional studies. In the in vitro test, both IAPs inhibited the growth of *Xanthomonas axonopodis* pv. *glycines*, which causes bacterial pustule disease in soybean [147]. In the *ex vivo* test, both IAPs alleviated the infection of the soybean leaves by *Phakopsora pachyrhizi* [147]. Furthermore, transgenic soybean plants expressing either IAP had enhanced resistance against Asian rust caused by *P. Pachyrhizi* [147].

In mouse, it was found that the intestinal α-defensin is regulated by matrilysin, a metalloproteinase (MMP) that was demonstrated to be able to cleave the precursor of α-defensin (also called cryptdin) in vitro to activate the antibacterial property of α-defensin, which is to disrupt the bacterial cell membrane [148]. Matrilysin-deficient mice were more susceptible to bacterial diseases [148]. In plants, *MMP* cDNAs have been cloned from *G. max* [149], *A. thaliana* [150], and *Cucumis sativus* [151]. In addition, MMP proteins have been purified from *Fagopyrum esculentum* [152] and *G. max* [153]. In soybean, it was found that the expression of *GmMMP2* was induced by the introduction of *PsgA* or *PsgC* (effectors from P. syringae pv. glycinea) into soybean cell suspension cultures and the metalloproteinase activity of GmMMP2 was also increased in these cell cultures [154]. The expression of *GmMMP2* was also induced by *P. sojae* infection, wounding, or dehydration of the plant [154].

Members of pathogenesis-related protein group 5 (PR-5) have been identified in soybean. In plants, the secretory PR-5 proteins could be induced by biotic/abiotic stresses [155,156,157]. Three isoforms of the *Glycine max* osmotin-like protein (GmOLP), GmOLPa [155], GmOLPb [157], and GmOLPc [156], which are the acidic, neutral, and alkaline forms of this PR-5 protein, respectively, have been characterized in soybean. The expression of *GmOLPa* was shown to be induced by salt stress, dehydration, or abscisic acid (ABA) [155]. Similarly, the expression of *GmOLPb* was shown to be induced by salt stress [157]. However, *GmOLPc* expression was found to be related to pathogens [156]. The GmOLPc molecule possesses an extended negatively charged cleft which enables the endo-(1,3)-β-D-glucanase activity. GmOLPc could bind to (1,3)-β-D-glucans and vesicles composed of 1,2-dipalmitoyl-*sn*-glycero-3-[phospho-*rac*-(1-glycerol)] (DPPG) [156]. Such binding and cleaving properties contribute to the antimicrobial activity of GmOLPc. GmOLPc purified from soybean hull could inhibit the growth of *Pseudomonas syringae* pv. *glycinea* and the germination of the spores of *Phytophthora sojae* [156].

By analyzing the differentially expressed genes during *P. sojae* infection of the soybean plant, an expressed sequence tag (EST) homolog of *Pru ar 1*, which is the major allergen in apricot (*Prunus armeniaca*) was found to be up-regulated in the resistant soybean cultivar, Suinong 10, upon infection [158]. Later, it was found that the full-length sequence of the EST encodes a 157-amino acid peptide, namely Gly m 4l [159]. The transcript level of *Gly m 4l* was highly upregulated by *P. sojae* infection, salicylic acid (SA) treatment, and NaCl treatment [159]. The Gly m 4l recombinant protein was shown to have RNase activity, and the ability to inhibit the growth of *P. sojae* and decrease the amount of *P. sojae* zoospores in vitro [159].

In an RNA-seq analysis of *G. max* nodules resulting from inoculation with *Bradyrhizobium japonium* strain 113-2, 60 cysteine-rich genes were found to be differentially expressed in nodules at different developmental stages [129]. Among these genes, nine of them were annotated as secretory proteins [129]. These cysteine-rich genes and their predicted functions are listed in Table 4. These NCR-like peptides share similar properties with NCR peptides found in other legumes. These properties include expression in nodules, being cysteine-rich and secretory. Although the functions of these proteins have yet to be elucidated, this finding hinted at the possibility that such cysteine-rich secretory proteins may have similar functions to those of the better-studied NRC peptides in other legumes.

### 7.2. AMPs Secreted by Soybean-Associated Microbes

The soybean plant not only encodes AMPs in its own genome to defend against pathogens, but it also recruits beneficial microbes that secrete AMPs for extra protection. These soybean-associated microbes are summarized in Table 5.

#### 7.2.1. Endophytes

Soybean endophytes have been reported to secrete antimicrobial peptides. *Paenibacillus* sp. strain HKA-15 is an endophyte isolated from soybean nodule [21]. The supernatant of a *Paenibacillus* sp. strain HKA-15 culture was found to inhibit the growth of *Rhizoctonia bataticola*, which causes charcoal rot in soybean by producing antifungal peptides [22]. *Paenibacillus* sp. HKA-15 improved the germination rate of *R. bataticola*-treated soybean seeds and reduced the disease incidence of the seedings [22]. The protective effect of *Paenibacillus* sp. HKA-15 on soybean was further demonstrated by *Paenibacillus* sp. HKA-15 mutants, which were unable to produce the antifungal peptides [22]. These mutants could not inhibit the growth of *R. bataticola*, nor protect soybean seeds and the germinated seedlings from *R. bataticola* infection [22]. When treated with the cell-free extract of *Paenibacillus* sp. HKA-15, the cytoplasm of *R. bataticola* cells showed abnormal contraction followed by cell lysis [22]. In a screen of soybean endophytic bacteria, 13 bacteria species/strains with antimicrobial properties were isolated from various tissues, including the root, stem, and leaf, of wild-type soybean or glyphosate-resistant transgenic soybean [23]. All of the 13 endophytic bacteria could inhibit the growth of *Sclerotinia sclerotiorum*, which causes white mold on soybean [23]. The detailed inhibitory targets are summarized in Table 5. Among the 13 endophytic bacteria, the methanol extract and ammonium sulfate precipitate of *Bacillus* sp. could inhibit the growth of *Sclerotinia sclerotiorum*, *Phomopsis sojae*, *Rhizoctonia solani*, 61*Xag* (*Xanthomonas axonopodis* pv. *glycines*), 62*Xag*, and *Pseudomonas savastanoi* pv. *glycinea* (*Psg*), although the microbe *Bacillus* sp. could not inhibit the growth of *Psg* in the in vitro antagonistic activity study [23]. Methanol extraction and ammonium sulfate precipitation are known methods of extracting peptides from the cell-free supernatant of bacterial cultures. Although methanol is also commonly used for secondary metabolite precipitation, the methanol extracts and ammonium sulfate precipitates exhibited similar antimicrobial effects [23]. Therefore, it was suggested that the antimicrobial activities of the endophytic bacteria are mainly due to secretory peptides [23].

#### 7.2.2. Rhizospheric Microbes

Certain microbes isolated from the rhizosphere of soybean have been found to exhibit antimicrobial functions via secretory peptides. In a search of soybean rhizospheric *Pseudomonas* and *Bacillus* spp. exhibiting antifungal activities, *Bacillus amyloliquefaciens* BNM340 was shown to inhibit the growth of ascomycetes including *Macrophomina phaseolina* BNM401 and *Sclerotinia minor* BNM402; mitosporic fungi including *Fusarium oxysporum* BNM403, *F. oxysporum* BNM404, *F. solani* BNM400, *F. solani* BNM405, and *F. solani* BNM406; and the oomycete, *Pythium ultimum* BNM407 [24]. *B. amyloliquefaciens* BNM340 could colonize soybean seeds and roots [24]. Further analyses showed that surfactin and iturin A were present in the cell-free supernatant of the *B. amyloliquefaciens* BNM340 culture [24]. Surfactin and iturin A are lipopeptides, and surfactin was found to have a synergistic effect on iturin A-induced hemolysis [25]. In another study, *Paenibacillus polymyxa* BRF-1 was isolated from the soybean rhizosphere. It was found to be able to protect soybean roots from rot disease [26]. Later, it was found that the extracellular metabolite filtrate of *P. polymyxa BRF-1* could inhibit the growth of *Rhizoctonia solani*, and the antifungal substance in the metabolite filtrate was identified as a 35.4-kDa peptide [27].

The findings from these previous reports suggest that AMPs produced by soybean and soybean-associated microbes have target-specificity, so not all pathogens could be inhibited by any one particular AMP. However, the detailed mechanism of the specificity is yet to be delineated. Importantly, many of the antimicrobial peptides from soybean and soybean-associated microbes have been shown to have antimicrobial activities in vitro. Such properties make the external application of soybean AMPs feasible for pathogen control, which will be discussed in Section 8.

## 8. The Potential Application of Soybean Antimicrobial Peptides and Soybean-Associated Microbes as Biopesticides

Synthetic chemicals have been the major components in pesticides for the control of plant pathogens [161]. However, the use of chemical pesticides poses negative impacts on the environment and human health [162]. Furthermore, the absorption and translocation of a pesticide by plants influences the efficacy of pesticide application [161]. On the other hand, the use of biopesticides is an environmentally friendly strategy for sustainable agriculture. Currently, several biopesticides are commercially available and have been summarized in previous reviews [163,164]. The active ingredients of these biopesticides include microbes such as fungi, oomycetes, and bacteria and many of the targets of these biopesticides are nematodes and insects [163]. However, considering the negative impacts brought forth by pathogenic microbes on soybean as discussed in Section 1, there is a need to develop biopesticides to protect soybeans from the pathogenic microbes. AMPs from soybean, which have been demonstrated to possess in vitro antimicrobial activities, and those produced by soybean-associated microbes are potential candidates for biopesticides against soybean diseases. As discussed in Section 7, these AMPs have target specificities and therefore, should cause minimal harm to non-target microbes. Thus, more research on AMPs produced by soybean and its associated microbes will be beneficial in facilitating the development of soybean biopesticides.

## 9. Conclusions

Immunity is a dynamic process that involves the secretion of effectors by pathogens and the resulting responses of plants, including the induction of defense-related genes to achieve resistance. Plants have evolved to recognize pathogens. Meanwhile, pathogens have evolved to evade detection by host plants. For example, soybean has successfully evolved to recognize flg22 from *R. solanacearum*, which causes bacterial wilt disease. This is a unique characteristic of soybean as *R. solanacearum* is virulent in more than 250 plant species. Soybean plants, soybean endophytes, and soybean rhizospheric microbes can also secrete antimicrobial peptides to defend against pathogens. These antimicrobial peptides and beneficial microbes are thus, potential active ingredients for soybean biopesticide production.

## Figures and Tables

**Table 1:** Common soybean diseases caused by fungi, oomycetes, bacteria, or viruses.

Disease	Pathogen	Microbe Type
Anthracnose	*Colletotrichum* spp.	Fungus
Brown stem rot	*Cadophora gregata* (Allington and D.W. Chamb.) T.C. Harr. and McNew (syn. *Phialophora gregata*)	
Charcoal rot	*Macrophomina phaseolina* (Tassi) Goid	
Downy mildew	*Pernospora manshurica* (Naumov) Syd. ex Gäum	
Foliar blight	*Rhizoctania solani* J.G. Kühn	
Frogeye leaf spot	*Cercospora sojina* Hara	
Northern stem canker	*Diaporthe phaseolorum* var. *caulivora* Athow and Caldwell	
Phomopsis seed decay	*Phomopsis* spp./*Diaporthe* spp.	
Pod and stem blight	*Diaporthe phaseolorum* var. *sojae* (Lehman) Wehm.	
Purple seed stain and Cercospora leaf blight	*Cercospora kikuchii* (Tak. Matsumoto and Tomoy.) M.W. Gardner	
Rust	*Phakopsora pachyrhizi* Syd. and P. Syd.	
Sclerotinia stem rot	*Sclerotinia sclerotiorum* (Lib.) de Bary	
Septoria brown spot	*Septoria glycines* Hemmi	
Sudden death syndrome	*Fusarium virguliforme* O’Donnell and T. Aoki, 2003	
Target leaf spot	*Corynespora cassiicola* (Berk. and M.A. Curtis) C.T. Wei	
Phytophthora root and stem rot and damping-off of seedlings	*Phytophthora sojae* Kaufm. and Gerd.	Oomycete
Damping-off of seedlings	*Pythium* spp. Pringsh.	
Downy mildew	*Peronospora manshurica* Syd. (Naumov)	
Damping off and root rot	*Pythium ultimum* Trow, 1901	
Seed rot	*Phytopythium* spp.	
Bacterial blight	*Pseudomonas syringae* pv. *glycinea* (Coerper 1919) Young et al., 1978	Bacterium
Bacterial pustule	*Xanthomona campestris* subsp. *glycines* (Nakano) Dye	
Bacterial tan spot	*Curtobacterium flaccumfaciens* pv. *flaccumfaciens* (Hedges 1922) Collins and Jones 1983	
Bacterial wilt	*Ralstonia solanacearum* Yabuuchi et al., 1996 (Smith, 1896)	
Fasciation	*Rhodococcus facians* (Tilford 1936) Goodfellow 1984	
Wildfire	*Pseudomonas syringae* pv. *tabaci* (Wolf and Foster, 1917) Young et al., 1978	
Bean pod mottle	Bean pod mottle virus	Virus
Bud blight	Tobacco ringspot virus	
Mosaic	Soybean mosaic virus Gardner and Kendrick (1921)	
Soybean vein necrosis virus	Soybean vein necrosis virus	
Yellow mosaic	Bean yellow mosaic potyvirus	

**Table 2:** Examples of *Phytophthora* spp. effector peptides and the associated infection mechanisms.

*Phytophthora* spp.	Effector Peptide	Host Target	Virulence Promotion Mechanism in Host	Reference
***P. sojae***	PsAvh23	ADA2 subunit of the ADA2/GCN5 module, part of the SAGA histone acetyltransferase (HAT) complex	Modulation of soybean H3K9 HAT by competitively binding to its regulatory subunit ADA2, preventing the association of catalytic subunit GCN5, thereby suppressing the activation of defense genes.	[15]
	PsAvh52	Putative transacetylase protein (GmTAP1)	Relocation of GmTAP1 to the nucleus, where it acetylates core histones to upregulate plant susceptibility genes.	[115]
	PsAvr3c	Serine/lysine/arginine-rich proteins (GmSKRP1/2) associated with spliceosome components	Stabilizes GmSKRP1, preventing its degradation. This leads to changes in host pre-mRNA splicing that ultimately lead to impaired plant immunity.	[117]
	PsAvh238	Type 2 1-aminocyclopropane-1-carboxylate synthase (Type 2 GmACS)	Suppression of ethylene synthesis by interacting with key biosynthesis enzyme Type 2 GmACS to promote infection.	[120]
	PsAvh262	Luminal binding immunoglobulin proteins (BiPs)	Stabilizes luminal binding BiPs of the endoplasmic reticulum (ER)-to suppress ER stress-triggered cell death and promote infection.	[121]
***P. infestans***	PITG_22798	Direct target still unknown	Transient expression in *Nicotiana benthamiana* showed nucleus localization and triggered cell death. The host avirulence effector 3b (AVR3b) suppressed PITG_22798-induced cell death.	[122]
	Pi17316	A Yeast-2-Hybrid screen proposed interaction with the potato ortholog of the putative MAP3K VASCULAR HIGHWAY 1-interacting kinase (StVIK).	Pi17316 putatively acts in the StVIK signal transduction pathway to modulate plant immunity. More detailed studies are needed.	[123]
***P. capsici***	PcAvh1	Putatively interacts with the scaffolding subunit of protein phosphatase 2A (PP2Aa)	Interferes with pathways regulating plant immunity and growth. More detailed studies are needed.	[124]
***P. parasitica***	PPTG00121 (= PpE4)	Direct target still unknown	PpE4 is necessary for full virulence of *P. parasitica*, but further studies are needed to comprehend its mode of action.	[125]
	PpRxLR2	Direct target still unknown	Transient expression experiments in *N. benthamiana* showed the capacity of PpRxLR2 to suppress programmed cell death in cells challenged with the elicitin INF-1.	[126]

**Table 3:** Characterized antimicrobial peptides from soybean.

Peptide	Peptide Activities	Reference
Gm0025x00667(75–100)	Growth inhibition of *Xanthomonas axonopodis* pv. *glycines* Alleviation of soybean leaves infection by *Phakopsora pachyrhizi*	[147]
Gm0026x00785(77–103)	Growth inhibition of *X. axonopodis* pv. *glycines*, Alleviation of soybean leaves infection by *P. pachyrhizi*	[147]
GmOLPc	Growth inhibition of *Pseudomonas syringae* pv. *glycinea*, Germination inhibition of the spores of *Phytophthora sojae*	[156]
Gly m 4l	RNase activity Growth inhibition of *P. sojae* Decrease of the amount of *P. sojae* zoospores	[159]

**Table 4:** NCR-like proteins in soybean and their predicted functions.

Gene ID ^#^	Predicted Functions ^^^	Expression Patterns ^^^
Glyma.05G235200	Stress response and antifungal	High expression in pods, seeds, and stems, relatively low in nodules
Glyma.08G042600	Stress response and antifungal	High expression in stems, flowers, and leaves, relatively low in nodules
Glyma.09G223500	Related to cell division	High expression in root hairs and shoot tips, relatively low in nodules
Glyma.10G133900	Stress response and antifungal	High expression in roots and unopen flowers, relatively low in nodules
Glyma.13G094100	Pathogenesis-related	High expression in nodules
Glyma.14G213600	Stress response and antifungal	High expression in root hairs and nodules
Glyma.18G040800	Stress response and antifungal	High expression in roots, stems, nodules
Glyma.19G168000	Stress response and antifungal	High expression in nodules
Glyma.20G200200	Stress response and antifungal	High expression in nodules

^#^ The gene list was retrieved from a soybean nodule RNA-seq analysis [129]. The gene IDs were converted to the soybean genome annotation version 2 format by Phytozome 12 [160]. ^^^ Prediction retrieved from Phytozome 12 [160].

**Table 5:** Soybean-associated microbes that exhibit antimicrobial activities.

Association with Soybean Plant	Type of Microbe	Symbiotic Tissue	Strain	Target Microbe(s)	Reference(s)
Endophytic	Bacterium	Nodule	*Paenibacillus* sp. HKA-15	*Rhizoctonia bataticola*	[21,22]
		Root	*Enterobacter ludwigii* (ID 226)	*Sclerotinia* *sclerotiorum*, *61Xag*	[23]
		Root	*Enterobacter* sp. (ID 231)	*Sclerotinia* *sclerotiorum*	
		Root	*Enterobacter* sp. (ID 219)	*Sclerotinia* *sclerotiorum*	
		Stem	*Agrobacterium* *tumefaciens*/*Rhizobium* sp. (ID 179)	*Sclerotinia* *sclerotiorum*	
		Leaf	*Kosakonia cowardii* (ID 79)	*Sclerotinia* *sclerotiorum*	
		Root	*Variovorax* sp. (ID 41)	*Sclerotinia* *sclerotiorum*	
		Stem	*Bacillus* sp. (ID 152)	*Sclerotinia* *sclerotiorum*	
		Root	*Burkholderia* sp. (ID 137)	*Sclerotinia* *sclerotiorum*, *Pseudomonas* *sojae*, *Rhizoctonia* *solani*	
		Root	*Burkholderia* sp. (ID 130)	*Sclerotinia* *sclerotiorum*, *Rhizoctonia* *solani*	
		Root	*Burkholderia* sp. (ID 243)	*Sclerotinia* *sclerotiorum*, *Pseudomonas* *sojae*	
		Leaf	*Pantoea vagans* (ID 106)	*Sclerotinia* *sclerotiorum*	
		Leaf	*Serratia marcescens* (ID 245)	*Sclerotinia* *sclerotiorum*	
		Root	*Enterobacter* sp. (ID 110)	*Sclerotinia* *sclerotiorum*	
Rhizospheric			*Bacillus amyloliquefaciens* BNM340	Ascomycota including *Macrophomina phaseolina* BNM401 and *Sclerotinia minor* BNM402 Mitosporic fungi including *Fusarium oxysporum* BNM403, *Fusarium oxysporum* BNM404, *Fusarium solani* BNM400, *Fusarium solani* BNM405, and *Fusarium solani* BNM406, and Oomycota *Pythium ultimum* BNM407	[24]
			*Paenibacillus polymyxa* BRF-1	*Rhizoctonia solani*	[27]
